# Supplementary material for: Brain and Muscle Oxygen Saturation Combined with Kidney Injury Biomarkers Predict Cardiac Surgery Related Acute Kidney Injury
Source: Diagnostics (Basel). 2021 Aug 31;11(9):1591. doi: 10.3390/diagnostics11091591 (PMC8466978; doi:10.3390/diagnostics11091591)
Supplement: Supplementary file 1 [file diagnostics-11-01591-s001.zip › diagnostics-1315868-supplementary.pdf]

Supplementary Materials

Text S1: Anaesthetic management and postoperative sedation

All patients received 50 μg·kg^{-1} of lorazepam and 40 mg of omeprazole. Patients treated with beta-blockers before hospital admission were also administered 12.5 mg of metoprolol one hour before transport to the operating theatre. All patients received the same intravenous anaesthesia induction, including 200 μg of fentanyl (Fentanyl, WZF Polfa, Warsaw, Poland), 0.8–1.5 mg·kg^{-1} of propofol (Propofol 1% MCT/LCT Fresenius Kabi, Warsaw, Poland), and 0.5–1 mg·kg^{-1} of rocuronium bromide (Rokuronium Kabi, Fresenius Kabi, Warsaw, Poland) for muscle relaxation. After intubation, patients were ventilated with an air/oxygen mixture with the *Aisys CS² General Electric* anaesthesia apparatus in pressure-regulated volume control mode. The oxygen concentration after intubation was set to 50% and adjusted according to blood gas analysis and SpO2 to maintain SpO2 between 94 and 98%. The latter rocuronium bromide dose was followed by a continuous infusion at the dose of 0.3–0.6 mg·kg^{-1}h^{-1} until the sternum was closed. For intraoperative analgesia, patients were administered fentanyl continuous infusion to reach the cumulative dose of 20–30 mcg·kg^{-1} depending on the duration of surgery. Before CPB, anaesthesia was maintained with sevoflurane in a semi-closed circuit with fresh gas flow between 0.5 and 1.4 l min^{-1}. Sevoflurane concentration was adjusted to the patient's haemodynamic parameters and the depth of anaesthesia with the target range of 35–55. The depth of anaesthesia was assessed with a BIS 4 Electrode Sensor (*Covidien, Norwood, USA*) connected to the Philips InteliVue module (*Philips, Best, The Netherlands*). During and after CPB, the maintenance of anaesthesia was accomplished using continuous intravenous infusion of propofol 2–4 mg·kg^{-1}h^{-1} titrated to maintain BIS inside the target range. Before anaesthesia induction, patients received 500 mL of balanced electrolyte solution (Plasmalyte, Baxter, Poland; Venolyte or Optilyte, Fresenius Kabi, Warsaw, Poland). Further intraoperative fluids were administered at the discretion of the attending anaesthesiologist including, in most cases, an additional 500 mL balanced electrolyte solution, before the beginning of cardiopulmonary bypass and further crystalloid infusion, depending on fluid responsiveness and central venous pressure (CVP). The only colloid solutions used during the procedure was 500 mL of Gelofusine added to CPB priming and occasionally 5% albumin solution after weaning from CPB.

Dexamethasone (Dexaven, Jelfa; Poland) 0.5 mg/kg b.w. was administered before CPB for the reduction in the inflammatory response to CPB and 2 mg of H_1 receptor blocker – clemastine, to reduce the effects of histamine release resulting from protamine administration. All patients were administered 2 g of cefazoline (Tarfazolin, Polfa, Trachomin, Poland) for operative wound infection prophylaxis.

Patient monitoring included 3 leads ECG (II, III, V_4), direct measurement of arterial blood pressure, central venous pressure (CVP), arterial blood haemoglobin saturation with oxygen (SpO_2), core temperature measured in the oesophagus, and peripheral temperature measured at 3rd fingertip. Pressures were assessed with TruWave transducers (*Edwards Lifesciences, Irvine, USA*), positioned at the level of the right atrium.

Intraoperative fluid management was uniform and included 10–15 mL·kg^{-1} of balanced electrolyte solution (Plasmalyte, Baxter, Poland; Venolyte or Optilyte, Fresenius Kabi, Warsaw, Poland). Additional portions of crystalloids during and after CPB were administered at the discretion of the anaesthesiologist, based on arterial blood pressure, fluid responsiveness, and CVP. When mean arterial blood pressure fell below 60mmHg, norepinephrine, or other catecholamine infusions when indicated, was administered at the discretion of the anaesthesiologist.

After the end of surgery, patients were transported to the postoperative ICU under sedation with propofol at a dose of 1–1.5 mg·kg^{-1}h^{-1}, which was continued throughout the initial three hours after surgery. Patients were assessed for weaning from mechanical ventilation hourly by nursing staff and the attending intensivist. Postoperative analgesia included paracetamol iv., metamizole i.v. and morphine sulfas as i.v. boluses of 2.5–5 mg. After patients became responsive, further morphine administration was titrated, based on pain assessment, with a numeric analog scale.

Text S2: Surgical and cardiopulmonary bypass management

All surgical procedures were performed via median sternotomy. The extracorporeal circuit consisted of roller pump Stöckert S5 (Stöckert GmbH, Freiburg, Germany) and membrane oxygenator Capiox RX25R (Terumo, Ann Arbor, MI, USA). Non-pulsatile extracorporeal circulation was maintained with the flow of a calculated cardiac index of 2.4 $L \cdot min^{-1}$ m^{-2} of the body surface area, during normothermia, and reduced by 0.1 L/1 °C in patients operated under hypothermia (most commonly 32 °C). In all patients, an arterial cannula for CPB was placed in the ascending aorta. Venous blood for CPB was drained either from the right atrium or from superior and inferior vena cava, depending on the type of surgery. Cold crystalloid cardioplegia solution (Plegisol, Hospira Inc, Lake Forest, IL, USA) was used to provide myocardial protection against ischemia given as one single dose (20 $mL \cdot kg^{-1}$) and repeated after 2 h

Red blood cell concentrate was transfused whenever hemoglobin concentration decreased below 7 $g \cdot dL^{-1}$ while on CPB, and below 8 $g \cdot dL^{-1}$ after weaning from CPB. After weaning from CPB, norepinephrine infusion was titrated to keep mean arterial blood pressure above 65 mmHg after ensuring that the heart filling pressures were adequate. Any use of catecholamine infusion, including norepinephrine, if longer than 1 hour was reported as catecholamine use. Catecholamine doses were not reported.

Table S1. Correlations between blood NGAL, cystatin C and NIRS parameters.

	NGAL Before Surgery		NGAL 3 Hours after Surgery		Postoperative Cystatin C	
	**R**	**p**	**r**	**p**	**r**	**p**
**BEFORE CPB**						
$SomO_2$ before anesthesia induction	−0.249	0.008	−0.170	0.075	−0.279	0.006
$rScO_2$ before anesthesia induction	−0.205	0.031	−0.146	0.126	−0.262	0.010
$SomO_2$ directly before skin incision	−0.396	0.001	−0.314	0.001	−0.295	0.004
$rScO_2$ directly before skin incision	−0.190	0.046	−0.195	0.040	−0.186	0.069
$SomO_2$ after sternum opening	−0.455	0.001	−0.388	0.001	−0.298	0.003
$rScO_2$ after sternum opening	−0.261	0.006	−0.243	0.010	−0.321	0.001
**WITHIN CPB**						
$SomO_2$ 20′ after aortic cross-clamping	−0.366	0.001	−0.318	0.001	−0.155	0.131
$rScO_2$ 20′ after aortic cross-clamping	−0.207	0.029	−0.177	0.062	−0.094	0.358
$SomO_2$ 40′ after aortic cross-clamping	−0.343	0.001	−0.307	0.001	−0.219	0.041
$rScO_2$ 40′ after aortic cross-clamping	−0.117	0.218	−0.100	0.296	−0.053	0.605
$SomO_2$ 20′ after aortic cross-clamp removal	−0.199	0.036	−0.166	0.081	−0.141	0.170
$rScO_2$ 20′ after aortic cross-clamp removal	−0.121	0.203	−0.105	0.269	−0.135	0.732
**AFTER CPB**						
$SomO_2$ 20′ after CPB	−0.283	0.003	−0.269	0.005	−0.232	0.025
$rScO_2$ 20′ after CPB	−0.123	0.203	−0.131	0.174	−0.129	0.213
$SomO_2$ 40′ after CPB	−0.302	0.002	−0.293	0.003	−0.135	0.190
$rScO_2$ 40′ after CPB	−0.129	0.197	−0.149	0.136	−0.149	0.161
$SomO_2$ 60′ after CPB	−0.201	0.083	−0.181	0.117	−0.266	0.030
$rScO_2$ 60′ after CPB	−0.160	0.163	−0.165	0.152	−0.105	0.394

Table S2. Logistic regression analyses for blood NGAL, cystatin, and NIRS cut-off values adjusted to age, EUROScore, creatinine and hemoglobin before surgery, CPB and selected parameters related to surgery in CS-AKI prediction.

Parameters	OR (95% CI)	*p*
Age-adjusted OR for blood NGAL before surgery ≥ 91.5 ng/ml	8.70 (2.72–27.5)	0.001
EUROScore-adjusted OR for blood NGAL before surgery ≥ 91.5 ng/ml	8.45 (2.60–27.5)	0.001
Creatinine before surgery-adjusted OR for blood NGAL before surgery ≥ 91.5 ng/ml	7.75 (2.40–25.0)	0.001
Hemoglobin before surgery-adjusted OR for blood NGAL before surgery ≥ 91.5 ng/ml	6.83 (2.09–22.3)	0.001
CPB time-adjusted OR for blood NGAL before surgery ≥ 91.5 ng/ml	18.7 (4.36–79.6)	0.001
Aortic cross-clamp time-adjusted OR for blood NGAL before surgery ≥ 91.5 ng/ml	14.3 (3.82–53.6)	0.001
Time to extubation-adjusted OR for blood NGAL before surgery ≥ 91.5 ng/ml	9.16 (2.14–39.2)	0.003
3 valves surgery-adjusted OR for blood NGAL before surgery ≥ 91.5 ng/ml	9.02 (2.77–29.4)	0.001
Age-adjusted OR for blood NGAL 3h after surgery ≥ 140.5 ng/ml	16.6 (4.78–57.5)	0.001
EUROScore-adjusted OR for blood NGAL 3h after surgery ≥ 140.5 ng/ml	18.6 (5.25–65.7)	0.001
Creatinine before surgery-adjusted OR for blood NGAL 3h after surgery ≥ 140.5 ng/ml	13.3 (3.78–47.1)	0.001
Hemoglobin before surgery-adjusted OR for blood NGAL 3h after surgery ≥ 140.5 ng/ml	16.0 (4.57–55.3)	0.001
CPB time-adjusted OR for blood NGAL 3h after surgery ≥ 140.5 ng/ml	28.7 (6.99–117.5)	0.001
Aortic cross-clamp time-adjusted OR for blood NGAL 3h after surgery ≥ 140.5 ng/ml	32.1 (7.30–141.3)	0.001
Time to extubation-adjusted OR for blood NGAL 3h after surgery ≥ 140.5 ng/ml	13.9 (3.26–59.5)	0.001
3 valves surgery-adjusted OR for blood NGAL 3h after surgery ≥ 140.5 ng/ml	18.2 (5.21–63.6)	0.001
Age-adjusted OR for postoperative cystatin C ≥ 1.23 mg/L	99.7 (11.6–857.9)	0.001
EUROScore-adjusted OR for postoperative cystatin C ≥ 1.23 mg/L	105.2 (11.6–779.9)	0.001
Creatinine before surgery-adjusted OR for postoperative cystatin C ≥ 1.23 mg/L	89.7 (10.3–779.9)	0.001
Hemoglobin before surgery-adjusted OR for postoperative cystatin C ≥ 1.23 mg/L	98.9 (10.8–908.2)	0.001
CPB time-adjusted OR for postoperative cystatin C ≥ 1.23 mg/L	80.0 (9.28–690.3)	0.001
Aortic cross-clamp time-adjusted OR for postoperative cystatin C ≥ 1.23 mg/L	88.1 (10.1–773.1)	0.001
Time to extubation-adjusted OR for postoperative cystatin C ≥ 1.23 mg/L	63.6 (8.78–657.2)	0.001
3 valves surgery-adjusted OR for postoperative cystatin C ≥ 1.23 mg/L	91.8 (10.8–783.0)	0.001
Age-adjusted OR for $SomO_2$ 20′ after CPB ≤ 54.5%	7.05 (1.83–27.2)	0.005
EUROScore-adjusted OR for $SomO_2$ 20′ after CPB ≤ 54.5%	6.76 (1.73–26.4)	0.006
Creatinine before surgery-adjusted OR for $SomO_2$ 20′ after CPB ≤ 54.5%	6.41 (1.68–24/5)	0.007
Hemoglobin before surgery-adjusted OR for $SomO_2$ 20′ after CPB ≤ 54.5%	5.21 (1.34–20.3)	0.017
CPB time-adjusted OR for $SomO_2$ 20′ after CPB ≤ 54.5%	6.10 (1.53–24.4)	0.010
Aortic cross-clamp time-adjusted OR for $SomO_2$ 20′ after CPB ≤ 54.5%	6.03 (1.55–23.5)	0.010
Time to extubation-adjusted OR for $SomO_2$ 20′ after CPB ≤ 54.5%	14.7 (1.75–122.6)	0.013
3 valves surgery-adjusted OR for $SomO_2$ 20′ after CPB ≤ 54.5%	6.86 (1.78–26.4)	0.010
Age-adjusted OR for $rScO_2$ 20′ after CPB ≤ 62.5%	6.88 (1.46–32.4)	0.015
EUROScore-adjusted OR for $rScO_2$ 20′ after CPB ≤ 62.5%	5.83 (1.21–28.0)	0.028
Creatinine before surgery-adjusted OR for $rScO_2$ 20′ after CPB ≤ 62.5%	7.58 (1.60–35.8)	0.011
Hemoglobin before surgery-adjusted OR for $rScO_2$ 20′ after CPB ≤ 62.5%	8.19 (1.70–39.6)	0.009
CPB time-adjusted OR for $rScO_2$ 20′ after CPB ≤ 62.5%	19.1 (2.26–161.1)	0.007
Aortic cross-clamp time-adjusted OR for $rScO_2$ 20′ after CPB ≤ 62.5%	16.8 (2.07–136.4)	0.008
Time to extubation-adjusted OR for $rScO_2$ 20′ after CPB ≤ 62.5%	4.34 (1.12–16.0)	0.033
3 valves surgery-adjusted OR for $rScO_2$ 20′ after CPB ≤ 62.5%	8.79 (1.79–43.0)	0.007

Abbreviations: CS-AKI – cardiac surgery-associated acute kidney injury; CPB – cardio-pulmonary bypass; NGAL - neutrophil gelatinase-associated lipocalin; OR – Odds ratio; CI – confidence interval $rScO_2$ – regional cerebral oxygen saturation measured by near-infrared spectroscopy, $SomO_2$ – somatic oxygen saturation of thenar muscles measured by near-infrared spectroscopy.

Table S3. Demographic, clinical, and laboratory characteristics and data of cardiac surgery in the groups of patients according to LVEF.

	**LVEF 30–45%** *n* **= 26**	**LVEF > 45%** *n* **= 78**	*p*
**PREOPERATIVE CHARACTERISTICS**			
Age (years)	67 (63–74)	68 (60–75)	0.429
Male, *n* (%)	17 (65)	36 (45)	0.113
Coronary artery disease, *n* (%)	20 (77)	54 (69)	0.376
Arterial hypertension, *n* (%)	18 (69)	58 (74)	0.352
Diabetes mellitus, n (%)	8 (30)	28 (35)	0.978
EUROScore (logistic)	7.7 (4.5–16.0)	5.4 (2.6–10.1)	0.023
Creatinine (mg/dL)	1.02 (0.87–1.24)	0.91 (0.87–1.24)	0.018
Hemoglobin (g/dL)	13.4 (12.1–14.5)	13.6 (12.5–14.5)	0.388
Preoperative anemia *, *n* (%)	8 (31)	18 (23)	0.096
Leukocyte count (G/L)	7.79 (7.09–9.20)	7.00 (6.23–8.25)	0.027
Angiotensin -converting enzyme inhibitors/sartans in premedication	9 (34)	23 (29)	0.451
Statins in premedication	15 (58)	54 (68)	0.433
**INTRAOPERATIVE CHARACTERISTICS**			
Aortic valve surgery, *n* (%)	15 (58)	55 (69)	0.344
Mitral valve surgery, *n* (%)	10 (38)	20 (25)	0.214
Aortic and mitral valves surgery, *n* (%)	3 (17)	4 (4)	0.077
Ascending aorta surgery including Bentall operation *n* (%)	2 (8)	6 (7)	0.999
3 valves surgery, *n* (%)	5 (19)	6 (7)	0.409
Other surgery, *n* (%)	2 (7)	5 (6)	0.989
CPB time (min)	123 (96–169)	118 (99–150)	0.274
Aortic cross-clamp time (min)	81 (66–116)	82 (67–104)	0.374
**POSTOPERATIVE CHARACTERISTICS**			
AKI presence	4 (15)	14 (17)	0.556
Serum creatinine on the 1st day post-surgery (mg/dL)	1.05 (0.81–1.17)	0.94 (0.79–1.18)	0.205
Serum creatinine on the 2nd day post-surgery (mg/dL)	1.04 (0.79–1.18)	0.89 (0.75–1.15)	0.136
Serum creatinine on the 3rd day post-surgery (mg/dL)	0.94 (0.70–1.02)	0.80 (0.71–1.01)	0.159
Catecholamine infusion on the 1st day post-surgery *n* (%)	11 (42)	28 (36)	0.490
Catecholamine infusion on the 2nd day post-surgery *n* (%)	8 (31)	11 (14)	0.080
Catecholamine infusion on the 3rd day post-surgery *n* (%)	4 (16)	9 (12)	0.731
Diuresis on the 1st day post-surgery (mL)	2485 (2108–2660)	2290 (1935–2555)	0.127
Fluid balance on the 1st day post-surgery (mL)	−600 (−890–240)	−540 (−855–20)	0.389
Fluid balance on the 2nd day post-surgery (mL)	−190 (−1300–200)	0 (−923–457)	0.221
Fluid balance on the 3rd day post-surgery (mL)	−100 (−700–200)	−325 (−875–200)	0.290
Postoperative chest drainage on the 1st day post-surgery (mL)	410 (243–690)	330 (240–575)	0.247
Postoperative chest drainage on the 2nd day post-surgery (mL)	200 (140–260)	155 (103–245)	0.248
Furosemide on the 1st day post-surgery, *n* (%)	14 (50)	45 (57)	0.456
Time to extubation (hours)	8.0 (7.0–12.0)	8.0 (6.0–10.0)	0.212
CRP on the 1st day post-surgery	27.0 (19.6–37.8)	30.7 (17.3–44.5)	0.316
CRP on the 2nd day post-surgery	73.3 (52.8–92.7)	68.8 (43.7–96.2)	0.467
CRP on the 3rd day post-surgery	117.0 (80.9–140.0)	95.3 (60.9–145.8)	0.366
WBC on the 1st day post-surgery	13.5 (11.7–16.4)	12.4 (10.2–14.7)	0.033
WBC in the 2nd day post-surgery	14.4 (13.7–17.5)	13.6 (11.3–15.7)	0.044
WBC in the 3rd day post-surgery	11.6 (8.3–13.1)	10.0 (7.8–12.3)	0.058
Hemoglobin on the 1st day post-surgery	10.6 (9.9–11.2)	10.6 (10.0–11.3)	0.271
Hemoglobin on the 2nd day post-surgery	9.7 (9.4–10.7)	10.2 (9.5–10.6)	0.361
Hemoglobin on the 3rd day post-surgery	9.2 (8.5–10.0)	9.8 (8.9–10.4)	0.069
**BIOMARKERS**			
Blood NGAL before surgery (ng/mL)	78.5 (68.5–130.8)	60.5 (48.0–91.3)	0.011
Blood NGAL 3 hours after surgery (ng/mL)	94.5 (59.5–129.0)	73.0 (51.0–98.3)	0.040
Postoperative cystatin C (mg/L)	0.99 (0.77–1.21)	0.89 (0.75–1.24)	0.250
**NIRS PARAMETERS**			
$SomO_2$ before anesthesia induction (%)	47.5 (41.8–57.3)	58.0 (50.0–65.0)	0.001
$SomO_2$ directly before skin incision (%)	58.5 (50.3–63.8)	64.0 (57.0–68.0)	0.038

$SomO_2$ after sternum opening (%)	53.0 (48.0–58.5)	58.0 (53.5–66.0)	0.010
$SomO_2$ 20′ after aortic cross-clamping (%)	45.0 (35.8–51.5)	51.0 (44.8–56.5)	0.006
$SomO_2$ 40′ after aortic cross-clamping (%)	42.5 (34.0–52.0)	49.0 (44.8–58.3)	0.008
$SomO_2$ 20′ after aortic cross-clamp removal (%)	53.5 (48.8–59.8)	60.5 (53.8–69.0)	0.003
$SomO_2$ 20′ after CPB (%)	49.0 (44.0–57.0)	56.0 (52.0–67.0)	0.001
$SomO_2$ 40′ after CPB (%)	48.0 (43.8–53.8)	55.5 (51.0–64.0)	0.001
$SomO_2$ 60′ after CPB (%)	53.0 (43.0–58.0)	57.0 (51.0–66.0)	0.016
$rScO_2$ before anesthesia induction (%)	57.5 (54.0–63.0)	63.0 (57.0–70.0)	0.046
$rScO_2$ directly before skin incision (%)	63.5 (53.3–69.8)	67.0 (62.0–74.0)	0.033
$rScO_2$ after sternum opening (%)	63.5 (57.0–68.0)	64.0 (58.8–71.0)	0.140
$rScO_2$ 20′ after aortic cross-clamping (%)	55.0 (51.3–61.8)	56.0 (50.0–62.0)	0.479
$rScO_2$ 40′ after aortic cross-clamping (%)	54.0 (48.0–61.0)	54.0 (47.0–60.0)	0.328
$rScO_2$ 20′ after aortic cross-clamp removal (%)	60.5 (52.0–67.8)	61.0 (55.0–67.0)	0.492
$rScO_2$ 20′ after CPB (%)	61.0 (57.0–66.0)	61.0 (55.0–67.0)	0.478
$rScO_2$ 40′ after CPB (%)	61.0 (56.0–70.0)	62.5 (56.8–69.0)	0.460
$rScO_2$ 60′ after CPB (%)	61.0 (58.0–67.0)	63.5 (57.3–70.0)	0.259

Data are presented as median (25th–75th percentyl) or numbers (and percent). Abbreviations: AKI—acute kidney injury; CPB—cardio-pulmonary bypass; CRP—C-reactive protein; LVEF—left ventricle ejection fraction; NGAL—neutrophil gelatinase-associated lipocalin; $rScO_2$—regional cerebral oxygen saturation measured by near-infrared spectroscopy, $SomO_2$—somatic oxygen saturation of thenar muscles measured by near-infrared spectroscopy; WBC—white blood cell. *—Preoperative anemia was defined as hemoglobin level <13 $g{\cdot}dL^{-1}$ in men and <12 $g{\cdot}dL^{-1}$ in women.
